# Control de la tuberculosis multirresistente a fármacos: un objetivo posible

**DOI:** 10.7705/biomedica.5216

**Published:** 2019-09-01

**Authors:** Jaime Robledo

**Affiliations:** 1 Unidad de Bacteriología y Micobacterias, Corporación para Investigaciones Biológicas, Medellín, Colombia; Escuela de Ciencias de la Salud, Universidad Pontificia Bolivariana, Medellín, Colombia Universidad Pontificia Bolivariana Escuela de Ciencias de la Salud Universidad Pontificia Bolivariana Medellín Colombia

La tuberculosis multirresistente es un obstáculo real para alcanzar el control de la enfermedad en el 2030, tal como lo establecen los objetivos de desarrollo sostenible ^1^. La proyección de la situación actual al 2050, permite visualizar que el 25 % de las muertes atribuidas a la resistencia a los medicamentos serán por la tuberculosis y que Mycobacterium tuberculosis será el agente patógeno respiratorio con las mayores cifras de resistencia ^2^. Además, y no menos importante para los programas de salud pública y la economía de los países, el costo económico de una muerte por tuberculosis multirresistente fluctúa entre USD$ 5.000 y USD$ 55.000 ^3^. Frente a estas cifras, resulta paradójico que se cuente con los medios de diagnóstico, tratamiento y prevención para impedir este posible desenlace en el futuro.

El desarrollo de nuevas tecnologías moleculares para establecer la sensibilidad a los medicamentos, como el Xpert para la rifampicina y los ensayos de sondas en línea para la isoniacida y la rifampicina, permite obtener resultados en horas, en comparación con los días que tardan los métodos microbiológicos basados en cultivos. Por ello, la Organización Mundial de la Salud (OMS) los ha avalado ^4^^,^^5^. El acceso universal a pruebas de sensibilidad y medicamentos de segunda línea puede disminuir la mortalidad en 73 % y la incidencia en 43 % en los próximos 30 años, según un estudio basado en un modelo (modelling approach) llevado a cabo en Moldavia, un país con alta prevalencia de tuberculosis multirresistente ^6^. 

La implementación de estas tecnologías requiere conocer su desempeño, establecer los valores predictivos de la sensibilidad a medicamentos en el contexto de la población estudiada, calcular el tiempo que tardan en generar un resultado y la eficacia de su implementación, con el fin de evitar que el diagnóstico no se mejore y se incrementen los costos de la atención, así como sincronizar la obtención de los resultados con el comienzo oportuno de un tratamiento efectivo. 

Los tratamientos de la tuberculosis multirresistente incluyen medicamentos más costosos que causan más reacciones secundarias, deben emplearse durante un tiempo más prolongado, y son menos efectivos y más costosos que los usados para la tuberculosis sensible a los fármacos. Una consecuencia directa de ello es el deficiente cumplimiento del tratamiento, con el consecuente aumento de la morbilidad y la mortalidad, el riesgo de desarrollar formas más resistentes, como la tuberculosis extremadamente resistente (TB-XDR), y la potencial transmisión de estas cepas a la comunidad. En la más reciente guía de la OMS para el tratamiento de la tuberculosis resistente a los fármacos se recomiendan nuevos medicamentos, se sugiere evitar otros por sus efectos tóxicos (inyectables, aminoglucósidos) y utilizar esquemas más cortos (9 a 12 meses en lugar de 18 a 20 meses) en los pacientes que cumplan con ciertas condiciones ^7^. Todavía es temprano para determinar a mediano y largo plazo el impacto de los nuevos medicamentos y de los tratamientos más cortos; sin embargo, un estudio en el que se evaluaron el resultado y los efectos secundarios de un tratamiento estandarizado de 9 meses en países de África, Report ó 72,4 % de curación ^8^, que está muy por encima de la obtenida con los tratamientos convencionales más prolongados. 

Los avances en el diagnóstico y en el tratamiento hacen parte de la solución para mejorar el control de la tuberculosis multirresistente; sin embargo, no serán suficientes para alcanzarlo si no se tienen en cuenta otros factores en el manejo de una enfermedad compleja y de amplio impacto social como la tuberculosis y sus formas resistentes a fármacos. 

Entender por qué un paciente llega a desarrollar una forma resistente de la enfermedad y determinar los factores de riesgo es indispensable para diseñar estrategias de prevención y control más efectivas. En una revisión sistemática y metaanálisis reciente de estudios adelantados en varias regiones del mundo, se establecieron como los factores de riesgo más importantes el haber sido tratado por tuberculosis previamente, el tener una edad mayor de 40 años, el estar desempleado, el no tener seguro de salud, el presentar baciloscopia positiva, efectos secundarios del tratamiento, la infección por HIV o por enfermedad pulmonar obstructiva crónica, el incumplir el tratamiento y el estar infectado por cepas del tipo Beijing; asimismo, algunos factores se asociaron con regiones geográficas específicas. Los autores concluyeron que, además de abordar factores de riesgo universales como la enfermedad y el tratamiento previo, son necesarios estudios regionales sobre factores de riesgo específicos para diseñar estrategias efectivas de control ^9^.

Es necesario determinar, no solo los factores de riesgo, incluidos algunos de los condicionantes sociales de la enfermedad, sino que es imprescindible facilitar el acceso al tratamiento y la comunicación entre el personal de salud y los enfermos, así como garantizar la protección social de los pacientes con tuberculosis multirresistente que incluya el apoyo emocional, social y económico ^10^.

En el 2017 la OMS estimó que en Colombia se habían dado 420 casos de tuberculosis multirresistente a la rifampicina ^11^, de los cuales se diagnosticaron menos del 60 % ^12^. Aunque el programa nacional de tuberculosis se ha fortalecido con su red de laboratorios, lo cual ha mejorado la accesibilidad a las pruebas de sensibilidad a los fármacos ^12^, la brecha entre los casos estimados y los reportados es uno de los retos que debe priorizarse.

Sin embargo, no basta con hacer el diagnóstico, sino que, además de hacerlo, se le debe proveer al paciente el mejor tratamiento posible, algo que el país está todavía en mora de solucionar. Los lineamientos técnicos y operativos del programa nacional de tuberculosis próximos a ser publicados abordan el uso de nuevos fármacos y de tratamientos más cortos para la tuberculosis multirresistente, acordes con las más recientes guías de la OMS. Se espera que no haya limitaciones futuras de acceso y disponibilidad del tratamiento para su implementación, como ha ocurrido en el pasado.

La incorporación de nuevas tecnologías diagnósticas y de nuevos fármacos y esquemas de tratamiento basados en la mejor información disponible, así como la comprensión del entorno individual y social del paciente con tuberculosis multirresistente, son retos que debe abordar el programa nacional de tuberculosis para garantizar que el diagnóstico oportuno y el tratamiento efectivo sean la mejor manera de controlar esta forma de la enfermedad en el futuro.
